# Automated ASPECTS Segmentation and Scoring Tool: a Method Tailored for a Colombian Telestroke Network

**DOI:** 10.1007/s10278-024-01258-9

**Published:** 2024-09-16

**Authors:** Esteban Ortiz, Juan Rivera, Manuel Granja, Nelson Agudelo, Marcela Hernández Hoyos, Antonio Salazar

**Affiliations:** 1 Systems and Computing Engineering Department, Universidad de los Andes, Bogotá, Colombia; 2 Department of Diagnostic Imaging, University Hospital Fundación Santa Fe de Bogotá, Bogotá, Colombia; 3 Grupo Suomaya, Servicio Nacional de Aprendizaje (SENA), Bogotá, Colombia; 4 Electrophysiology and Telemedicine Laboratory, Universidad de los Andes, Bogotá, Colombia

**Keywords:** Telestroke, ASPECTS, Stroke, Cerebrovascular disease, Software, Algorithms, Emergency medicine, Segmentation, Thrombolysis

## Abstract

To evaluate our two non-machine learning (non-ML)-based algorithmic approaches for detecting early ischemic infarcts on brain CT images of patients with acute ischemic stroke symptoms, tailored to our local population, to be incorporated in our telestroke software. One-hundred and thirteen acute stroke patients, excluding hemorrhagic, subacute, and chronic patients, with accessible brain CT images were divided into calibration and test sets. The gold standard was determined through consensus among three neuroradiologist. Four neuroradiologist independently reported Alberta Stroke Program Early CT Scores (ASPECTSs). ASPECTSs were also obtained using a commercial ML solution (CMLS), and our two methods, namely the Mean Hounsfield Unit (HU) relative difference (RELDIF) and the density distribution equivalence test (DDET), which used statistical analyze the of the HUs of each region and its contralateral side. Automated segmentation was perfect for cortical regions, while minimal adjustment was required for basal ganglia regions. For dichotomized-ASPECTSs (ASPECTS < 6) in the test set, the area under the receiver operating characteristic curve (AUC) was 0.85 for the DDET method, 0.84 for the RELDIF approach, 0.64 for the CMLS, and ranged from 0.71–0.89 for the neuroradiologist. The accuracy was 0.85 for the DDET method, 0.88 for the RELDIF approach, and was ranged from 0.83 − 0.96 for the neuroradiologist. Equivalence at a margin of 5% was documented among the DDET, RELDIF, and gold standard on mean ASPECTSs. Noninferiority tests of the AUC and accuracy of infarct detection revealed similarities between both DDET and RELDIF, and the CMLS, and with at least one neuroradiologist. The alignment of our methods with the evaluations of neuroradiologist and the CMLS indicates the potential of our methods to serve as supportive tools in clinical settings, facilitating prompt and accurate stroke diagnosis, especially in health care settings, such as Colombia, where neuroradiologist are limited.

## Introduction

Acute stroke is a significant cause of mortality and morbidity in both developed [1–3] and undeveloped countries [4]. Stroke episodes can manifest as either ischemic or hemorrhagic, necessitating precise diagnostic evaluation through imaging expertise prior to initiating treatment [5]. Surgical intervention may be considered in select cases of hemorrhagic stroke, whereas acute reperfusion therapies, such as thrombolytic therapy and endovascular thrombectomy, are viable options for managing ischemic stroke [5].

Recent large core trials, including SELECT 2, RESCUE-Japan, and ANGEL ASPECT, have demonstrated that among individuals with severe ischemic strokes, endovascular thrombectomy yields superior functional outcomes compared to those achieved with standard medical care, albeit with an association with vascular complications. Noncontrast head computed tomography (NCCT) serves as the primary imaging modality for promptly detecting both hemorrhagic and ischemic strokes in patients presenting with acute neurological deficits [6–8]. Ischemic infarct size within middle cerebral artery (MCA) territory was estimated with the Alberta Stroke Program Early CT Score (ASPECTS) [9]. The ASPECTS is associated with the features of ten regions in the vascular territory of the middle cerebral artery MCA: six cortical areas and four basal ganglia regions [9]. Typically, a patient with an ASPECTS < 6, as determined through nonadvanced imaging techniques under usual circumstances, is not eligible for acute reperfusion therapies) [6, 7, 10, 11].

The advancement of optimal machine learning technologies in the context of stroke is a complex undertaking contingent upon the utilization of extensive and high-quality datasets. The accessibility of such datasets is frequently constrained by factors such as the absence of data sharing agreements, apprehensions regarding patient privacy, and the substantial expenses associated with data sharing, storage, and quality control. These challenges consistently impede the establishment of robust open-access stroke registries [12, 13]. A significant limitation to the progress of machine learning-based algorithms in stroke treatment lies in the inherent difficulties required for follow-up assessments, which are particularly pronounced in developing countries where the absence of consistent and robust health systems hampers sustained long-term follow-up. Several researchers, conducting performance analyses and comparisons of diverse machine learning algorithms, have noted that approximately fifty percent of participants were excluded from their analyses due to missing outcomes and predictive variables. This exclusion introduces a level of uncertainty into the results [14].

ASPECTSs can be calculated by a specialized neuroradiologist; nevertheless, when someone with such expertise is not available in person or by teleradiology networks, an automated ASPECTS calculation may provide helpful information for neuroradiologists or neurologists during patient evaluation. However, methods based on AI require a large amount of data for tuning or optimization of the algorithms [15]. Commercial solutions that include automated ASPECTS modules [16] can be expensive, there is limited information on how they process the information and were not tailored to our local population.

The automated calculation of the ASPECTS involves two primary steps: segmenting the ASPECTS regions and subsequently evaluating the presence or not of an ischemic infarct within each region before computing the overall score. ASPECTS regions can be automatically identified using standardized coordinate spaces for patient volume alignment [17, 18], convolutional neural networks (CNNs) [19], registration algorithms using a reference template or an atlas [20–23], and deep learning networks (DLNs) [24, 25]. Methods for ischemic infarct evaluation using artificial intelligence (AI) are split into automatic algorithms using DLNs [25, 26] or CNNs [22–24], and explicit feature-based methods focusing on intensity or texture. Intensity features include the average and standard deviation (SD) [27]; the brain density shift comparing intensity histograms [20, 21]; and pixel intensity differences [17, 18, 27]. These features are compared against thresholds, contralateral regions, or used in binary classifiers [18, 28, 29]. Unfortunately, those AI methods require large amounts of well-labeled datasets, which are scarce for acute ischemic stroke [15].

The aim of this study was to develop and to validate the diagnostic performance two non-ML-based methods to detect ischemic infarcts in ASPECTS-identified regions tailored to the local population; these methods will be integrated into our web-based telestroke network system [30]. To avoid the need for large datasets of AI methods for ischemic infarct evaluation, statistical parameters of the intensities ASPECTS regions, measured in Hounsfield Units (HUs), were evaluated. In addition, patients involved in the study for tunning the algorithms were issued of our local population. Hence, a low-cost and tailored solution was achieved.

## Materials and Methods

Our institutional review board (IRB) approved this retrospective study and waived the requirement for informed consent.

### Study Design and Dataset

Our hospital is a private primary stroke center certified by the Joint Commission International and is one of the few centers in Colombia with 24/7 endovascular thrombectomy capabilities. Patients with acute stroke symptoms who presented to our hospital between 2013 and 2018 and for whom the stroke code was activated were eligible for the study. Patients were randomly selected without repetition. The diagnostic NCCT images were acquired using a standard protocol with a General Electric LightSpeed 64 slice CT scanner (General Electric Healthcare, GE Medical Systems, Milwaukee, WI, USA) with the following parameters: 100 kV, 10 mA, axial: 5 mm, sagittal: 3 mm, FOV: 26 cm, pixel spacing: 0.5 × 0.5 mm, and matrix: 512 × 512. The images were stored in standard Digital Imaging and Communication in Medicine (DICOM) format. Initially, CT images with artifacts and patients younger than 18 years were excluded.

This was a repeated measures study. All NCCT images were interpreted by four neuroradiologist, three with more than ten years of experience and one with four years of experience in neuroradiology, who reported the presence of acute ischemic infarcts and calculated the ASPECTS for each patient. The ASPECTS for each patient was also obtained using three automated ASPECTS systems: a commercial machine learning solution (CMLS), i.e., RapidAI ASPECTS (iSchema View, Menlo Park, CA, USA) [31], and the two algorithms proposed in this study.

Neuroradiologist interpretations were carried out using a DICOM-compliant medical workstation. The medical workstation used the viewer software Agfa IMPAX 6.5 (AGFA HealthCare, Mortsel, Belgium). Images were displayed using an E-2620 BARCO monitor (BARCO N. V, Kortrijk, Belgium), which has a 2-megapixel (MPx) LCD medical grayscale display, is DICOM-compliant, has a dot pitch of 0.249 mm, has a spatial resolution of 1600 × 1200 pixels, has a maximum luminance of 700 cd/m2, and displays 8-bit grayscale images. Relevant clinical data, such as sex, age, neurological symptoms, and medical history (e.g., diabetes, hypertension, and cardiac arrhythmia), were available to a neuroradiologist. We then compared the neuroradiologist’ interpretations to the results of the automated methods.

A detailed description of the sample, observers, reading systems, interpretation procedure, and data acquisition approach used in the present evaluation was reported in previous studies, in which the diagnostic performance of readers using different display systems was evaluated﻿ [32–34]. At the time of these studies, no automated ASPECTSs were available in our system.

### Gold Standard

The true status of the presence of ischemic infarcts in the ASPECTS-identified regions was established by three more experienced neuroradiologist at our hospital (with 2/3 of the neuroradiologist in agreement). Our routine practice is to perform a follow-up CT scan 24 h after the initial CT scan. Therefore, neuroradiologist reviewed the initial and 24-h follow-up CT images, following the gold standard. In patients who experienced infarct evolution in the first 24 h, we could not perform a follow-up CT scan because the treatment decision was based on the initial CT scan.

### Image Standardization

The initial processing of the images involved intensity normalization, where high- and low-intensity values were clipped to a predefined range. Additionally, noise reduction processes [35] were employed to enhance image clarity. Only pixels with intensities ranged from 10–55 HU were considered, to avoid cerebrospinal fluid and chronic infarcts, bone, and calcifications. For skull stripping, our algorithm first identified skull regions based on intensity thresholds. The largest skull contour was detected [36], and a mask was applied to isolate the brain area, effectively removing the skull from the image. The cerebral area was then trimmed by creating a mask to distinguish the brain area from the background, and the image was cropped to focus on the region of interest [37]. Postprocessing included resizing images to a standard dimension and adding uniform space around the image to maintain the brain's central position [37].

### Image Analysis and Processing

**Fig. 1:**
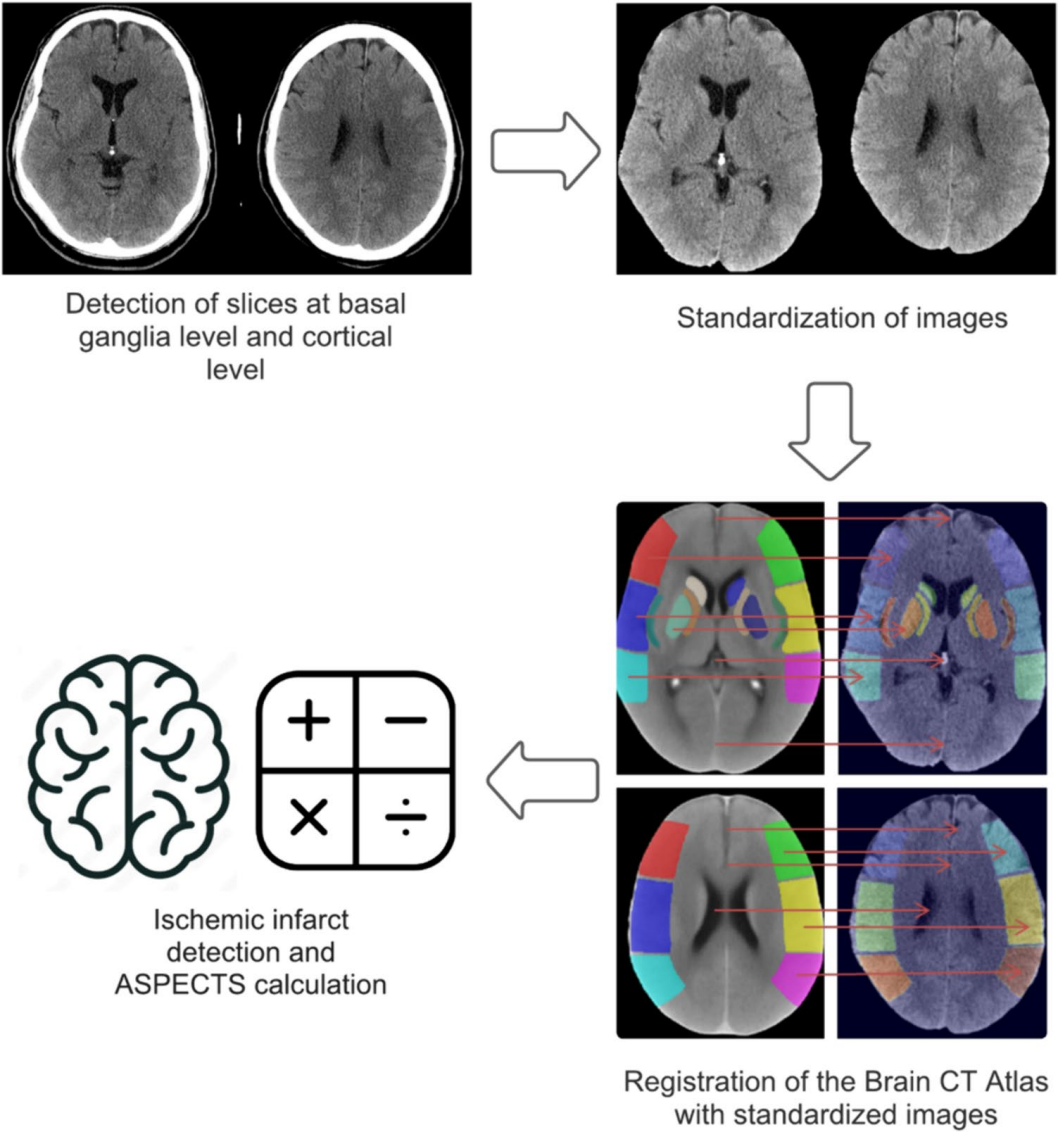
Operational flow of the proposed algorithm

Our methodology integrates advanced imaging and machine learning techniques to assess the impact of ischemic stroke within the MCA of the brain. For this algorithm, the first step is to standardize brain CT images using the ASPECTS-identified regions, focusing on segmentation of the basal ganglia and cortical regions. This process involves employing a CT-brain atlas [38] for alignment through both rigid and nonrigid registration techniques [39]. Our approach includes the use of a CNN to classify the images into distinct categories. Each step of our methodology, from image processing to the application of machine learning models, is designed to provide a comprehensive assessment of ischemic stroke effects, contributing to improved diagnostic and treatment strategies. Figure 1 summarizes the operational flowchart of our proposed algorithm.

### Detection of Slices at the Basal Ganglia and Cortical Levels

The first step in segmentation was the detection of two slices, at the basal ganglia and cortical levels, in which ASPECTS-related regions must be identified. We employed a CNN utilizing transfer learning, specifically adopting the InceptionResNetV2 architecture [40] with pretrained ImageNet weights, excluding the top layer. Additional layers included convolutional, max pooling, flattening, and dense layers, culminating with an activation function for classification.

### Registration of the Brain CT Atlas with Standardized Images

Rigid registration involved thresholding both atlas slices and patient images to enhance structural visibility, applying a 2D similarity transformation model for alignment [41], and resampling the atlas images [42] for initial overlay. Nonrigid registration starts with an identity transformation [43] and is optimized using a mutual information metric [44]. Additional image processing techniques, such as dilatation of specific regions [45], were used to enhance the visibility and distinction of important anatomical features. The atlas was resampled again, considering the nonrigid transformation, for precise overlay, with a specific focus on internal basal ganglia regions.

### Detection of Ischemic Infarcts

For metric calculation, the mean intensity difference between corresponding regions in the left and right hemispheres was calculated within specific HU ranges [45] to target relevant tissue densities. The relative hemispheric difference was also computed as a measure of asymmetry in tissue density. In the region-specific analysis, regions of interest were extracted from preprocessed images for detailed analysis, including the calculation of the mean and SD of the HU intensity for each region.

Two methods for automatic ischemic infarct detection that do not use AI were proposed in this pilot study.

#### Mean HU relative difference (RELDIF)

This method calculates the relative difference Δ of the mean HU as a percentage of the hypodensity observed between a region and its contralateral side. If the relative difference Δ is greater than or equal to a calibration threshold, the region is considered to have an ischemic infarct. For each ASPECTS region, the calibration threshold was determined by calculating the area under the receiver operating characteristic (ROC) curve (AUC) for a large range of thresholds (0.05–50%, with steps of 0.05%), and the threshold producing the maximum AUC, with a specificity of at least 0.88, was selected as the calibration threshold for the region. This method was named RELDIF. A similar method was used in a previous study [46].

#### Density Distribution Equivalence Test (DDET)

This method is based on statistical Z test comparisons of two means with different standard deviations. In this method, the mean and standard error (SE) of a region were calculated over the HU of all the pixels in the region. Then an equivalence test is performed between the mean HU of the right and left regions. If significant results allowed us to claim equivalence of the two regions, no ischemic infarct was determined. In contrast, if nonequivalence was observed, a superiority test was performed to determine the region with the ischemic infarct. In the equivalence test, the null hypothesis was |difference (I-J)|—δ = 0, and the alternative hypothesis was |difference (I-J)|—δ < 0, where I and J are the mean HUs of the compared regions and δ is an HU margin representing the maximum difference indicating equivalence [47–50]. To find the optimal δm for each region, the AUC for a large range of HU margins (0.05–16 HU, with steps of 0.05 HU) and the margin producing the maximum AUC, with a specificity of at least 0.88, were selected as the calibration margins for the region. This method was named DDET. A method comparing the contralateral mean and SD was proposed by Shieh Y et al. [27], but margin thresholds were not used, and they are used in the present study.

In accordance with actual clinical procedures, neuroradiologist were provided with information regarding the side (laterality) of the focal acute neurological deficit prior to interpreting head CT images. Subsequently, either the DDET or RELDIF methods were utilized to assess the presence of a lesion within each ASPECTS-identified region on the affected hemisphere. Conversely, the CMLS (machine learning system) does not utilize these data; therefore, determination of the lesion side is solely based on imaging analysis and a training dataset.

### Variables

The “Ischemic infarct detected” variable for the right and left hemispheres of each ASPECTS-identified region was established using two proposed methods (DDET and RELDIF). Thereafter, using the “Ischemic infarct detected” variable, two derived variables were calculated: 1) the ASPECTS for each patient and 2) the “Dichotomized-ASPECTS”, indicating that the ASPECTS was less than 6 (ASPECTS < 6), which was the main variable in our evaluation, as this was the main imaging contraindication for IV r-TPA administration.

### Statistical Analysis

To evaluate and compare the diagnostic performance of our automatic ASPECTS calculation methods, the CMLS, and four neuroradiologists’ readings, several validity indicators were calculated for the three variables defined above, including nonparametric receiver operating characteristic (ROC) curves and the AUC, accuracy, specificity, and sensitivity. To calculate the AUC of each rater and the differences between two raters, DBM-MRMC 2.51 software (Medical Image Perception Laboratory, Iowa University, USA) was used [51]. Sensitivity, specificity, and accuracy were evaluated using generalized estimating equations (GEEs) in IBM SPSS Statistics 29 software (IBM Corp., Armonk, NY, USA); this software was also used to evaluate reliability with the Kappa coefficient [52]. The Kappa coefficients were ranked as defined by Altman [53]: “very good”, (1–0.81); “good”, (0.8–0.61); “moderate”, (0.6–0.41); “fair”, (0.4–0.21); and “poor”, < 0.2.

Noninferiority tests were performed for the AUC, accuracy, specificity, and sensitivity using the mean differences of paired comparisons and their SEs. The null hypothesis for the noninferiority test was difference (I-J) = -δ, and the alternative hypothesis was difference (I-J) >—δ, where I and J are the compared systems and δ is the maximum difference permitted to claim noninferiority, while for the equivalence test, the null hypothesis was |difference (I-J)|—δ = 0, and the alternative hypothesis was |difference (I-J)|—δ < 0 [47–50]. The significance level was set to α = 0.05.

## Results

To determine the validity of our proposed methods for automatic ischemic infarct detection, optimized for our local population, we aimed to evaluate its diagnostic performance by comparing its validity indicators, as AUC, accuracy, specificity, sensitivity, between them and against the four neuroradiologist and the CMLS.

There was a total of 188 patients for whom the stroke code was activated and were eligible. Among these 188 patients, those with hemorrhagic (n = 25), subacute (n = 31), and chronic (n = 19) stroke were excluded, resulting in a final sample of 113 patients. Patients were aged 30 to 94 years, with a mean age of 69.7 years (SD = 15.1), and 58 (51%) were males.

The 113 patients were separated into two sets, a calibration set (n = 65) used to find the optimized parameters required in our automatic ASPECTS calculations. The second set (n = 48) was used to test and compare our automatic ASPECTSs with those calculated by the four neuroradiologists and the CMLS.

According to the gold standard, there were 52 patients with nonvisible ischemic infarcts (i.e., ASPECTS = 10) and 66 with ASPECTSs < 10. At basal ganglia regions, the most frequent infarcts were at the internal capsule (49), and at cortical regions the most frequent infarcts were at the MCA cortex lateral to insular ribbon (Table 1).

**Table 1:** Distribution of infarcts according to the gold standard

ASPECTS Region	Negative	Positive	Total
C: Caudate head	103	10	113
I: Insular ribbon	64	49	113
IC: Internal capsule	108	5	113
L: Lentiform nucleus	93	20	113
M1: Anterior MCA cortex	91	22	113
M2: MCA cortex lateral to insular ribbon	78	35	113
M3: Posterior MCA cortex	99	14	113
M4: Anterior MCA territories	87	26	113
M5: Lateral MCA territories	82	31	113
M6: Posterior MCA territories	99	14	113

*ASPECTS* Alberta Stroke Program Early CT Score, *MCA* middle cerebral artery

### Detection of Slices at the Basal Ganglia and Cortical Levels

A dataset of 1484 brain CT slices (from 85/113 patients) was categorized into three classes: basal ganglia, cortical, and control. This dataset was partitioned such that 80% went into the training set and 20% went into validation set. Model training over 50 epochs resulted in a loss of 18%, a precision (VPP) of 93.7%, and a recall (sensitivity) of 93.3% during training and a loss of 25.6%, a precision of 92.9%, and a recall of 92.9% during validation.

### ASPECTS- Regions Identification

Within the two slices detected, the contours of the ASPECTS-identified regions were plotted, and a neuroradiologist established whether the contours required additional adjustment before evaluating the presence of an ischemic infarct. The quality results of this segmentation for cortical regions showed a perfect match, with no adjustments needed in 100% of regions, while at basal ganglia regions, it ranged from 85 to 96% (Table 2).

**Table 2:** Segmentation Results

ASPECTS region	Quality of segmentation^a^
C: Caudate head	75/85 (88%)
I: Insular ribbon	73/85 (85%)
IC: Internal capsule	79/85 (92%)
L: Lentiform nucleus	82/85 (96%)
M1: Anterior MCA cortex	85/85 (100%)
M2: MCA cortex lateral to insular ribbon	85/85 (100%)
M3: Posterior MCA cortex	85/85 (100%)
M4: Anterior MCA territories	85/85 (100%)
M5: Lateral MCA territories	85/85 (100%)
M6: Posterior MCA territories	85/85 (100%)

^a^ Indicates that no image adjustment was needed

*ASPECTS* Alberta Stroke Program Early CT Score, *MCA* middle cerebral artery

### Optimization of the Parameters for DDET and RELDIF Algorithms

The 65 patients of the calibration set were used to find the optimized parameters required in our automatic ASPECTS calculations. The equivalence margins δm parameter required for the DDET method were ranged from 1.95–3.9 HU at basal ganglia regions and ranged from 0.25–1.65 HU at cortical regions. The threshold Δ parameter for the RELDIF required for method were ranged from 4–9.1% at basal ganglia regions and ranged from 1.3–5.05% at cortical regions (Table 3). The AUC achieved with those parameters at the ten regions ranged from 0.57–0.86 for DDET, and higher values were observed for the accuracy, ranged from 0.83–0.94 for DDET, and ranged from 0.78–0.92 for RELDIF (Table 3).

**Table 3:** Equivalence margin δm and threshold Δ values for the maximum AUC in the calibration set

Density distribution equivalence test (DDET)						
ASPECTS region	δm (HU)^a^	Cases	AUC	Accuracy	Specificity	Sensitivity
C: Caudate head	3.9	65	0.57	0.91	0.98	0.17
I: Insular ribbon	2.05	65	0.71	0.77	0.88	0.55
IC: Internal capsule	1.75	65	0.70	0.89	0.90	0.50
L: Lentiform nucleus	1.95	65	0.86	0.92	0.95	0.78
M1: Anterior MCA cortex	0.75	65	0.70	0.83	0.89	0.50
M2: MCA cortex lateral to insular ribbon	0.7	65	0.71	0.78	0.89	0.53
M3: Posterior MCA cortex	0.95	65	0.77	0.86	0.88	0.67
M4: Anterior MCA territories	0.25	65	0.73	0.83	0.92	0.53
M5: Lateral MCA territories	0.8	65	0.74	0.83	0.90	0.57
M6: Posterior MCA territories	1.65	65	0.82	0.94	0.97	0.67
Mean HU relative difference (RELDIF)						
ASPECTS region	Δ Threshold (%)	Cases	AUC	Accuracy	Specificity	Sensitivity
C: Caudate head	9.1	65	0.57	0.91	0.98	0.17
I: Insular ribbon	4	65	0.76	0.80	0.88	0.64
IC: Internal capsule	4.85	65	0.72	0.92	0.94	0.50
L: Lentiform nucleus	4.9	65	0.85	0.91	0.93	0.78
M1: Anterior MCA cortex	1.55	65	0.70	0.83	0.89	0.50
M2: MCA cortex lateral to insular ribbon	1.3	65	0.71	0.78	0.89	0.53
M3: Posterior MCA cortex	5.05	65	0.73	0.92	0.97	0.50
M4: Anterior MCA territories	2.3	65	0.72	0.86	0.98	0.47
M5: Lateral MCA territories	1.8	65	0.74	0.83	0.90	0.57
M6: Posterior MCA territories	3.9	65	0.81	0.92	0.95	0.67

*ASPECTS* Alberta Stroke Program Early CT Score, *HU* Hounsfield Units, *AUC* area under ROC curve, *ROC* receiver operating characteristic, *MCA* middle cerebral artery

^a^ Margin in the paired Z test to indicate equivalence of the HU density distribution of a region on both hemispheres

### Overall ASPECTS

The mean ASPECTS on the test set (n = 48) was 7.56 for the gold standard, 7.6 for RELDIF, and a lower mean of 7.4 was observed for DDET (Table 4). The mean ASPECTS for the other methods was greater, ranging from 8.23–8.67. Equivalence, at a margin of 5%, was documented among the gold standard, DDET, and RELDIF (all *P* < 0.001) (Table 4).

**Table 4:** Mean ASPECTS on the test set

Rater	Cases	Mean ASPECTS	SE	95% CI		*P* for equivalence test	
				LB	UB	DDET	RELDIF
Gold standard	48	7.56	0.36	6.83	8.29	< 0.001^a^	< 0.001^b^
DDET	48	7.40	0.38	6.63	8.16		< 0.001^b^
RELDIF	48	7.60	0.35	6.90	8.31	< 0.001^a^	
CMLS	48	8.67	0.28	8.10	9.23	0.16	0.41
Neuroradiologist 1	48	8.25	0.37	7.51	8.99	0.28	0.07
Neuroradiologist 2	48	8.67	0.29	8.09	9.25	0.17	0.40
Neuroradiologist 3	48	8.23	0.34	7.54	8.92	0.26	0.06
Neuroradiologist 4	48	8.46	0.31	7.83	9.09	0.42	0.29

*ASPECTS* Alberta Stroke Program Early CT Score, *SE* standard error of the mean, *CI* confidence interval, *LB* Lower bound, *UB* Upper bound, *HU* Hounsfield Units, *DDET* Density distribution equivalence test, *RELDIF* Mean HU relative difference

^a^ *P* value showing equivalence (*P* < 0.05) of the DDET compared to that of the second method at a margin of 5%

^b^ *P* value showing equivalence (*P* < 0.05) of the RELDIF compared to that of the second method at a margin of 5%

### Dichotomized-ASPECTS

Using the calibration parameters on the test set (n = 65), the AUC, sensitivity, specificity, and accuracy were calculated for the Dichotomized-ASPECTS with our two automatic methods (DDET and RELDIF). The mean AUCs were 0.82 for DDET and 0.84 for RELDIF and 0.64 for CMLS; the mean AUCs ranged from 0.71–0.89 for the neuroradiologists. The accuracy of DDET was 0.85 and 0.88 for RELDIF and ranged from 0.83–0.96 for the other raters. The specificity of DDET was 0.87 and 0.90 for RELDIF and ranged from 0.95–1.00 for the other raters. The sensitivity was 0.78 for DDET, 0.78 for RELDIF, and ranged from 0.33–0.78 for the other raters (Table 5).

**Table 5:** AUC for the Dichotomized-ASPECTS (ASPECTS < 6) in the test set

Rater	Cases	Mean	SE	95% CI		*P* for non-inferiority test	
				LB	UB	DDET	RELDIF
AUC							
DDET	48	0.82	0.082	0.66	0.99		0.15
RELDIF	48	0.84	0.081	0.68	1.00	0.21	
CMLS	48	0.64	0.089	0.47	0.82	0.006^a^	0.005^b^
Neuroradiologist 1	48	0.88	0.078	0.72	1.00	0.33	0.29
Neuroradiologist 2	48	0.72	0.092	0.54	0.90	0.04^a^	0.03^b^
Neuroradiologist 3	48	0.89	0.077	0.74	1.00	0.37	0.33
Neuroradiologist 4	48	0.71	0.093	0.53	0.89	0.03^a^	0.02^b^
Accuracy							
DDET	48	0.85	0.045	0.77	0.94		0.03^b^
RELDIF	48	0.88	0.045	0.79	0.96	0.11	
CMLS	48	0.83	0.045	0.75	0.92	0.03^a^	0.02^b^
Neuroradiologist 1	48	0.94	0.045	0.85	1.00	0.40	0.28
Neuroradiologist 2	48	0.90	0.045	0.81	0.98	0.18	0.11
Neuroradiologist 3	48	0.96	0.045	0.87	1.00	0.47	0.40
Neuroradiologist 4	48	0.88	0.045	0.79	0.96	0.11	0.06
Specificity							
DDET	48	0.87	0.033	0.81	0.94		0.005^b^
RELDIF	48	0.90	0.033	0.83	0.96	0.06	
CMLS	48	0.95	0.033	0.88	1.00	0.31	0.15
Neuroradiologist 1	48	0.97	0.033	0.91	1.00	0.50	0.31
Neuroradiologist 2	48	1.00	0.033	0.93	1.00	0.26	0.50
Neuroradiologist 3	48	1.00	0.033	0.93	1.00	0.26	0.50
Neuroradiologist 4	48	0.97	0.033	0.91	1.00	0.50	0.31
Sensitivity							
DDET	48	0.78	0.149	0.48	1.00		0.32
RELDIF	48	0.78	0.149	0.48	1.00	0.32	
CMLS	48	0.33	0.149	0.04	0.63	0.007^a^	0.007^b^
Neuroradiologist 1	48	0.78	0.149	0.48	1.00	0.32	0.32
Neuroradiologist 2	48	0.44	0.149	0.15	0.74	0.02^a^	0.02^b^
Neuroradiologist 3	48	0.78	0.149	0.48	1.00	0.32	0.32
Neuroradiologist 4	48	0.44	0.149	0.15	0.74	0.02^a^	0.02^b^

*ASPECTS* Alberta Stroke Program Early CT Score, *SE* standard error, *CI* confidence interval, *LB* Lower bound, *UB* Upper bound, *AUC* area under ROC curve, *ROC* receiver operating characteristic, *HU* Hounsfield Units, *DDET* Density distribution equivalence test, *RELDIF* Mean HU relative difference

^a^ *P* showing noninferiority (*P* < 0.05) of the DDET compared to that of the second method

^b^ *P* showing noninferiority (*P* < 0.05) of the RELDIF compared to that of the second method

To compare the performance of both the DDET and RELDIF methods, paired noninferiority statistical tests with a noninferiority margin of 0.1 were performed (Table 5). The DDET method confirmed the noninferiority of the AUC with respect to the CMLS (*P* = 0.006), to the Neuroradiologist 2 (*P* = 0.04), and to the Neuroradiologist 4 (*P* = 0.03). The RELDIF method confirmed the noninferiority of the AUC with respect to the CMLS (*P* = 0.005), to the Neuroradiologist 2 (*P* = 0.03), and to the Neuroradiologist 4 (*P* = 0.02). The DDET method confirmed the noninferiority of the sensitivity with respect to the CMLS (*P* = 0.007), to the Neuroradiologist 2 (*P* = 0.02), and to the Neuroradiologist 4 (*P* = 0.02). The RELDIF method confirmed the noninferiority of the AUC with respect to the CMLS (*P* = 0.007), to the Neuroradiologist 2 (*P* = 0.02), and to the Neuroradiologist 4 (*P* = 0.02). Noninferiority accuracy was confirmed between DDET versus CMLS (*P* = 0.03), and between RELDIF versus CMLS (*P* = 0.02). For specificity, noninferiority was observed for DDET versus RELDIF (*P* = 0.005).

### Detection of Ischemic Infarcts by Region

The AUC for all ten regions pooled was 0.7 for DDET, 0.71 for RELDIF, 0.66 for CMLS, and 0.71–0.8 for the neuroradiologists. The accuracy was 0.77 for DDET, 0.79 for RELDIF, and 0.81 for CMLS and ranged from 0.84–0.89 for the neuroradiologists. The specificity was 0.84 for the DDET and 0.86 for the RELDIF but ranged from 0.94–0.97 for the other methods. In contrast, the sensitivities of both the DDET and the RELDIF were 0.56 and 0.38 for the CMLS, respectively, and ranged from 0.44–0.63 for the neuroradiologists (Table 6).

**Table 6:** Overall lesion detection using the test set

Indicator	Rater	N	Mean	*P* for equivalence test		*P* for non-inferiority test		*P for superiority test*	
				DDET	RELDIF	DDET	RELDIF	DDET	RELDIF
AUC									
	DDET	480	0.70		0.004^b^		0.001^a^		0.40
	RELDIF	480	0.71	0.004^b^		0.004^a^		0.60	
	CMLS	480	0.66	0.04^b^	0.06	< 0.001^a^	< 0.001^a^	0.11	0.08
	Neuroradiologist 1	480	0.76	0.13	0.09	0.13	0.09	0.97	0.95
	Neuroradiologist 2	480	0.71	0.003^b^	0.003^b^	0.003^a^	0.002^a^	0.56	0.46
	Neuroradiologist 3	480	0.80	0.50	0.41	0.50	0.41	0.99	0.99
	Neuroradiologist 4	480	0.73	0.01^b^	0.008^b^	0.01^a^	0.008^a^	0.77	0.69
Accuracy									
	DDET	480	0.77		< 0.001^b^		< 0.001^a^		0.25
	RELDIF	480	0.79	< 0.001^b^		< 0.001^a^		0.75	
	CMLS	480	0.81	0.005^b^	< 0.001^b^	0.005^a^	< 0.001^a^	0.92	0.78
	Neuroradiologist 1	480	0.86	0.34	0.11	0.34	0.11	0.99	0.99
	Neuroradiologist 2	480	0.84	0.11	0.02^b^	0.11	0.02^a^	0.99	0.98
	Neuroradiologist 3	480	0.89	0.34	0.50	0.34	0.50	0.99	0.99
	Neuroradiologist 4	480	0.84	0.11	0.053	0.11	0.053	0.99	0.99
Specificity									
	DDET	480	0.84		< 0.001^b^		< 0.001^a^		0.10
	RELDIF	480	0.86	< 0.001^b^		< 0.001^a^		0.90	
	CMLS	480	0.94	0.30	0.15	0.30	0.15	0.99	0.99
	Neuroradiologist 1	480	0.95	0.15	0.30	0.15	0.30	0.99	0.99
	Neuroradiologist 2	480	0.97	0.06	0.50	0.06	0.50	0.99	0.99
	Neuroradiologist 3	480	0.97	0.06	0.50	0.06	0.50	0.99	0.99
	Neuroradiologist 4	480	0.96	0.15	0.30	0.15	0.30	0.99	0.99
Sensitivity									
	DDET	480	0.56		0.08		0.08		0.55
	RELDIF	480	0.56	0.08		0.049^a^		0.45	
	CMLS	480	0.38	0.08	0.11	< 0.001^a^	< 0.001^a^	0.003^c^	0.004^c^
	Neuroradiologist 1	480	0.57	0.08	0.08	0.08	0.99	0.55	0.60
	Neuroradiologist 2	480	0.44	0.38	0.38	< 0.001^a^	0.001^a^	0.03^c^	0.046^c^
	Neuroradiologist 3	480	0.63	0.30	0.30	0.30	0.34	0.84	0.87
	Neuroradiologist 4	480	0.50	0.31	0.31	0.006^a^	0.008^a^	0.15	0.18

*AUC* area under ROC curve, *ROC* receiver operating characteristic, *HU* Hounsfield Units, *DDET* Density distribution equivalence test, *RELDIF* Mean HU relative difference; N = 48 patients by 10 ASPECTS-regions

^a^ Confirmed noninferiority of our method compared with the second method at a margin of δ = 10%

^b^ Confirmed equivalence of our method compared with that of the second method at a margin of δ = 10%

^c^ Confirmed superiority of our method compared with that of the second method

For the AUC equivalence, at a margin of 0.1, was confirmed between the DDET and the RELDIF (*P* = 0.004), between the DDET and the CMLS (*P* = 0.04), between the Neuroradiologist 2 and both the DDET and the RELDIF (both *P* = 0.003), and between the Neuroradiologist 4 and the both the DDET (*P* = 0.01) and the RELDIF (*P* = 0.008); in addition, noninferiority was observed for the RELDIF against the CMLS (*P* < 0.001) (Table 6).

For the sensitivity superiority was confirmed for the DDET over the CMLS (*P* = 0.003), and for the RELDIF over CMLS (*P* = 0.004); in addition, superiority was confirmed for both the DDET and the RELDIF, over the Neuroradiologist 2 (*P* = 0.03 and *P* = 0.046 respectively). Other comparisons for accuracy, specificity, and sensitivity were included in Table 6. The detailed results grouped by basal ganglia regions (Table 7) and cortical regions were also evaluated (Table 8).

**Table 7:** Basal ganglia lesion detection using the test set

Indicator	Rater	N	Mean	*P* for equivalence test		*P* for non-inferiority test		*P* for superiority test	
				DDET	RELDIF	DDET	RELDIF	DDET	RELDIF
AUC									
	DDET	192	0.71		0.047^b^		0.047^a^		0.55
	RELDIF	192	0.70	0.047^b^		0.03^a^		0.45	
	CMLS	192	0.74	0.11	0.14	0.11	0.14	0.72	0.76
	Neuroradiologist 1	192	0.78	0.26	0.3	0.26	0.3	0.88	0.90
	Neuroradiologist 2	192	0.74	0.09	0.11	0.09	0.11	0.68	0.73
	Neuroradiologist 3	192	0.83	0.34	0.29	0.34	0.29	0.99	0.99
	Neuroradiologist 4	192	0.80	0.43	0.48	0.43	0.48	0.95	0.96
Accuracy									
	DDET	192	0.82		0.007^b^		0.007^a^		0.62
	RELDIF	192	0.81	0.007^b^		0.002^a^		0.38	
	CMLS	192	0.84	0.02^b^	0.04^b^	0.02^a^	0.04^a^	0.77	0.85
	Neuroradiologist 1	192	0.88	0.15	0.2	0.15	0.20	0.96	0.97
	Neuroradiologist 2	192	0.87	0.09	0.15	0.09	0.15	0.93	0.96
	Neuroradiologist 3	192	0.91	0.39	0.5	0.39	0.50	0.99	0.99
	Neuroradiologist 4	192	0.89	0.18	0.29	0.18	0.29	0.97	0.99
Specificity									
	DDET	192	0.91		< 0.001^b^		< 0.001^a^		0.70
	RELDIF	192	0.90	< 0.001^b^		< 0.001^a^		0.30	
	CMLS	192	0.93	0.002^b^	0.006^b^	0.002^a^	0.006^a^	0.79	0.91
	Neuroradiologist 1	192	0.97	0.06	0.12	0.06	0.12	0.99	0.99
	Neuroradiologist 2	192	0.99	0.12	0.35	0.12	0.35	0.99	0.99
	Neuroradiologist 3	192	0.98	0.12	0.22	0.12	0.22	0.99	0.99
	Neuroradiologist 4	192	0.96	0.02^b^	0.06	0.02^a^	0.06	0.97	0.99
Sensitivity									
	DDET	192	0.51		0.17		0.17		0.50
	RELDIF	192	0.51	0.17		0.17		0.50	
	CMLS	192	0.56	0.30	0.30	0.30	0.30	0.67	0.67
	Neuroradiologist 1	192	0.58	0.37	0.37	0.37	0.37	0.74	0.74
	Neuroradiologist 2	192	0.49	0.23	0.23	0.12	0.12	0.42	0.42
	Neuroradiologist 3	192	0.69	0.23	0.23	0.23	0.23	0.95	0.95
	Neuroradiologist 4	192	0.64	0.37	0.37	0.37	0.37	0.90	0.90

*AUC* area under ROC curve, *ROC* receiver operating characteristic, *HU* Hounsfield Units, *DDET* Density distribution equivalence test, *RELDIF* Mean HU relative difference; N = 48 patients by 4 ASPECTS-regions

^a^ Confirmed noninferiority of our method compared with the second method at a margin of δ = 10%

^b^ Confirmed equivalence of our method compared with the second method at a margin of δ = 10%

^c^ Confirmed superiority of our method compared with the second method

**Table 8:** Cortical lesion detection using the test set

Indicator	Rater	N	Mean	*P* for equivalence test		*P* for non-inferiority test		*P* for superiority test	
				DDET	RELDIF	DDET	RELDIF	DDET	RELDIF
AUC									
	DDET	288	0.69		0.03^b^		0.004^a^		0.34
	RELDIF	288	0.71	0.03^b^		0.03^a^		0.66	
	CMLS	288	0.61	0.35	0.48	< 0.001^a^	< 0.001^a^	0.03^c^	0.01^c^
	Neuroradiologist 1	288	0.75	0.2	0.1	0.2	0.1	0.92	0.84
	Neuroradiologist 2	288	0.69	0.02^b^	0.046^b^	0.009^a^	0.003^a^	0.44	0.28
	Neuroradiologist 3	288	0.78	0.39	0.24	0.39	0.24	0.98	0.94
	Neuroradiologist 4	288	0.68	0.03^b^	0.06	0.006^a^	0.002^a^	0.37	0.23
Accuracy									
	DDET	288	0.74		0.03^b^		< 0.001^a^		0.15
	RELDIF	288	0.77	0.03^b^		0.03^a^		0.85	
	CMLS	288	0.78	0.04^b^	0.003^b^	0.04^a^	0.003^a^	0.90	0.58
	Neuroradiologist 1	288	0.85	0.38	0.18	0.38	0.18	0.99	0.98
	Neuroradiologist 2	288	0.82	0.27	0.050	0.27	0.050	0.99	0.91
	Neuroradiologist 3	288	0.87	0.18	0.38	0.18	0.38	0.99	0.99
	Neuroradiologist 4	288	0.82	0.27	0.04^b^	0.27	0.04^a^	0.99	0.90
Specificity									
	DDET	288	0.79		0.04^b^		< 0.001^a^		0.03^c^
	RELDIF	288	0.84	0.04^b^		0.04^a^		0.97	
	CMLS	288	0.95	0.006^b^	0.23	0.006^a^	0.23	0.99	0.99
	Neuroradiologist 1	288	0.94	0.03^b^	0.5	0.03^a^	0.5	0.99	0.99
	Neuroradiologist 2	288	0.95	0.006^b^	0.23	0.006^a^	0.23	0.99	0.99
	Neuroradiologist 3	288	0.96	0.006^b^	0.23	0.006^a^	0.23	0.99	0.99
	Neuroradiologist 4	288	0.95	0.006^b^	0.23	0.006^a^	0.23	0.99	0.99
Sensitivity									
	DDET	288	0.60		0.15		0.15		0.57
	RELDIF	288	0.58	0.15		0.08		0.43	
	CMLS	288	0.26	0.003^b^	0.005^b^	< 0.001^a^	< 0.001^a^	< 0.001^c^	< 0.001^c^
	Neuroradiologist 1	288	0.57	0.19	0.15	0.06	0.08	0.37	0.43
	Neuroradiologist 2	288	0.42	0.16	0.20	< 0.001^a^	< 0.001^a^	0.02^c^	0.02^c^
	Neuroradiologist 3	288	0.59	0.13	0.13	0.10	0.13	0.47	0.54
	Neuroradiologist 4	288	0.40	0.14	0.16	< 0.001^a^	< 0.001^a^	0.01^c^	0.02^c^

*AUC* area under ROC curve, *ROC* receiver operating characteristic, *HU* Hounsfield Units, *DDET* Density distribution equivalence test, *RELDIF* Mean HU relative difference; N = 48 patients by 6 ASPECTS-regions

^a^ Confirmed non-inferiority of our method compared with the second method at a margin of δ = 10%

^b^ Confirmed equivalence of our method compared with the second method at a margin of δ = 10%

^c^ Confirmed superiority of our method compared with the second method

The overall agreement on infarcts detection between the DDET and RELDIF using the test set was “Almost perfect” (Kappa = 0.8); for basal ganglia regions pooled was “Almost perfect” (Kappa = 0.88), and for cortical regions pooled was “Substantial” (Kappa = 0.75). For individual regions, agreements ranged from 0.77–92, and for cortical regions were ranged from 0.38–1.0, with the lower value for M4- Anterior MCA territories (Table 9).

**Table 9:** Agreement on infarcts detection between the DDET and RELDIF using the test set

ASPECTS region		N	Kappa	SE	95% CI		Agreement
					LB	UB	
Overall		960	0.80	0.026	0.75	0.86	Very Good
Basal ganglia		384	0.88	0.034	0.81	0.94	Very Good
	I: Insular ribbon	96	0.92	0.044	0.84	1.00	Very Good
	IC: Internal capsule	96	0.91	0.055	0.80	1.00	Very Good
	L: Lentiform nucleus	96	0.77	0.109	0.56	0.98	Good
	L: Lentiform nucleus	96	0.82	0.090	0.65	1.00	Very Good
Cortical		576	0.75	0.037	0.68	0.82	Good
	M1: Anterior MCA cortex	96	0.94	0.055	0.84	1.05	Very Good
	M2: MCA cortex lateral to insular ribbon	96	1.00	0.000	1.00	1.00	Very Good
	M3: Posterior MCA cortex	96	0.50	0.095	0.32	0.69	Moderate
	M4: Anterior MCA territories	96	0.38	0.096	0.19	0.57	Fair
	M5: Lateral MCA territories	96	0.86	0.075	0.72	1.00	Very Good
	M6: Posterior MCA territories	96	0.78	0.095	0.59	0.96	Good

*HU* Hounsfield Units, *DDET* Density distribution equivalence test, *RELDIF* Mean HU relative difference; N = 48 patients by 2 raters by 4, 6 or 10 ASPECTS-regions. *SE* standard error, *CI* confidence interval, *LB* Lower bound, *UB* Upper bound

## Discussion

### Principal Results

Based on the detection of slices at the basal ganglia and cortical levels, high values for recall and precision were observed for the training and validation sets (all > 92%). Within these slices, a perfect match was observed (100%) in cortical ASPECTS-identified regions, and few adjustments were required for basal ganglia regions (matches ranged from 85–96%). For the Dichotomized-ASPECTS (ASPECTSs < 6), which is a contraindication to thrombolysis treatment, the mean AUCs observed using DDET (0.85) and RELDIF (0.88) were noninferior to that of two neuroradiologist, with a margin of 0.1. The detection of individual ischemic infarcts in the ASPECTS-identified regions showed higher sensitivity and lower specificity for our methods than for neuroradiologist, resulting in final ASPECTSs for DDET and RELDIF lower than those of neuroradiologist but statistically equivalent to the gold standard. Therefore, neuroradiologist and the CMLS showed a less conservative behavior, leading to more patients undergoing thrombolysis when assessed by the former than by our methods.

The detection of individual ischemic infarcts in the ASPECTS-identified regions showed higher sensitivity and lower specificity for our methods than for neuroradiologist, resulting in final ASPECTSs for DDET and RELDIF lower than those of neuroradiologist but statistically equivalent to the gold standard. The overall agreement on infarcts detection between the DDET and RELDIF shows their readability, allowing us to integrate either or both in our telestroke system.

### Comparison with Prior Work

Several studies have evaluated automated ASPECTSs. Wolf et al. evaluated the syngo.via Frontier ASPECTS software (Siemens Healthcare GmbH, Erlangen, Germany) [46], which is based on the relative density difference between the affected and contralateral regions and obtained a mean AUC of 0.713 for the detection of an affected ASPECTS region (overall ten regions). A similar study was performed by Ayobi et al. using CINA-ASPECTS software (Avicenna. AI, La Ciotat, France), which uses deep learning and obtains higher AUC, sensitivity, and specificity values. Nagel et al. evaluated e-ASPECTS software (Brainomix®, Oxford, UK) and concluded that e-ASPECTS was noninferior to neuroradiologist in determining ASPECTSs. However, those studies are not comparable to our study, as they calculated scores for both hemispheres as if they were from different patients (20 ASPECTS-identified regions, instead of 10 in our study).

Chen et al. [54] evaluated the overall incidence of ischemic infarcts in NBC (NeuBrainCARE) software (Neusoft Medical Systems, China) and RapidAI ASPECTS software (iSchema View, Menlo Park, CA, USA) [31], obtaining AUCs of 0.71 and 0.76 for NBC and the CMLS, respectively; however, this study included only patients who had already received intravenous thrombolysis or mechanical thrombectomy and did not compare dichotomized-ASPECTSs, as in our study. However, the study of Ferreti et al. [55], also conducted using e-ASPECTS, reported dichotomized-ASPECTS evaluations, and obtained a mean AUC for this variable of 0.78 and a sensitivity and specificity of 0.75 and 0.73, respectively; these values are lower than the means observed in our study for DDET (0.82, 0.78 and 0.87, respectively) and for RELDIF (0.84, 0.78 and 0.9, respectively).

### Limitations

A limitation of this pilot study is the lack of chronic patients. Even if these patients were included in our original neuroradiologists’ interpretation procedure, the ASPECTS regions were not evaluated for ischemic infarcts in these patients. Therefore, chronic patients were excluded from our comparisons. The same was true for subacute cases more than 4.5 h after onset, as those patients are also contraindicated for IV r-TPA. Nevertheless, if chronic lesions are included in our algorithms, its pixels are excluded as only those ranged from 10–55 HU are retained. Subacute lesions are not a problem either, as ischemic tissues exhibit decreased intensity over time [56], resulting in a more accurate detection. In addition, in our sample, there were few ischemic infarcts in the internal capsule or in the caudate head. Including more positive patients would improve the optimization of the margin for all regions, therefore improving the diagnostic performance of our methods. Our results promise statistically significant conclusions with lower margins and a small sample size, while AI methods require a greater sample size.

### Future Work

Our two methods were validated, so that all 113 patients could be used in the calibration process, instead of the initial 65, to improve the performance of the algorithms, as we have the gold standard, the neuroradiologists' interpretations, and the CMLS evaluations, for all these cases.

The evaluation of ischemic infarct detection was performed using the slices selection and region segmentation provided by automated algorithms; however, after integrating this tool into our telestroke software, neuroradiologists could select better slices or adjust the ASPECTS-detected regions if desired, hence improving the performance of our system.

The algorithms developed in this work could be used with other populations after appropriated calibration. In the first instance, the optimization of the algorithm parameters was carried out with patients from the capital of the country (8 million inhabitants). In order to generalize it to other populations, several hospitals in different regions of the country, including indigenous and Afro-descendant populations, will be included in a next phase of development. This will allow us to evaluate if the optimization can be done with the aggregate of patients or if an optimization is necessary for each group of patients, according to their ethnicity and origin. This would also be the case for applications in other countries, especially in Latin America, which do not have their own stroke management tools.

Finally, as we have the patients’ images of chronic and subacute patients, the gold standard for the lesions at the ASPECTS-regions will be the established, allowing to increase the sample size and to better calibrate the algorithms for those cases. Once this is done, it will be put into production as a plug-in to the image viewing tools of our telestroke system [30], which provides support for in situ patient management, advanced hospital referral recommendations, teleradiology and teleneurology. This software is the basis of a new development called “appremia®” [57] which allows among others, integration with geo-referenced ambulance networks, WhatsApp communications for notifications to specialists and patient's relatives, as well as specialist schedule management.

## Conclusions

The algorithm shows promise as a helpful diagnostic tool, particularly in settings, such as Colombia, where health care resources are limited. We were able to obtain an algorithm adjusted to the local population by calibrating it with our own patients.

The alignment of our methods with the evaluations of neuroradiologist indicates the potential of our methods to serve as supportive tools in clinical settings, facilitating prompt and accurate stroke diagnosis. This approach is crucial for early stroke detection, where timely interventions are vital for patient outcomes.

Additionally, the algorithm's consistent performance across various patient groups, including those not involved in the initial optimization, highlights its generalizability and applicability in real-world clinical scenarios. Our study thus offers significant insights into the application of machine learning in medical diagnostics, presenting a viable approach to stroke assessment and treatment in resource-limited environments.

## Data Availability

The data is stored in a private database and can be shared upon request.

## Abbreviations

AI: Artificial intelligence
ASPECTS: Alberta Stroke Program Early CT Score
AUC: Area under ROC curve
CI: Confidence interval
CMLS: Commercial machine-learning solution
CNN: Convolutional neural networks
DDET: Density Distribution Equivalence Test
DICOM: Digital Imaging and Communication in Medicine standard
DLN: Deep learning networks
HU: Hounsfield Units
LB: Lower bound
MCA: Middle cerebral artery
ML: Machine learning
NCCT: Non-contrast head computed tomography
RELDIF: Relative difference
ROC: Receiver operating characteristic
SD: Standard deviation
SE: Standard error
UB: Upper bound
